# Fat and Lean

**Published:** 1873-10

**Authors:** 


### FAT AND LEAN.

Meat eaters and vegetarians show in their persons the effects of the diet. The first has the most brain force and nervous energy. A mixed food of animal and vegetable rations developes the highest intellectual powers. A strictly vegetable living ordinarily gives a fair complexion and amiability, and extreme pugnacity when the vegetarian's views in regard to that one engrossing thought of his life are discussed. They are annual meeting reformers, without ever setting a river on fire. Arabs are a sober, frugal race, rather slender, not tall, conscientious and contentious on religious subjects. They largely subsist on rice, pulse, milk and keimac, something similar to whipped cream, through a vast region o an arid country where they are indigenous. They are not destitute of mutton, goats, camels and game; but they manifest no disposition to feed upon meats, as is necessary in temperate zones or in high northern latitudes. An intellectual man, one of their kindred, who rises to distinction by the grandeur of his mental status, is extremely rare. The beer and ale drinkers expand and grow fat, but they are not much given to profound researches in science.—*Med. & Sur. Reporter.*

